# Remarkable Change of Position of the Spleen and Abscess of the Same

**Published:** 1850-11

**Authors:** J. J. Lescher

**Affiliations:** Mt. Carmel, Ill.


### ARTICLE V.

*Remarkable change of position of the Spleen and abscess of the same.*—By J. J. Lescher, M.D., of Mt. Carmel, Ill.

Sometime in the spring of '48, in the early part of the evening, I was hastily called to visit a widow lady, some two miles distant, on the opposite side of the Wabash river, who, as the messenger reported, was suffering intensely from a tumor on the right and lower side of the abdomen. As speedily as I could, I reached her house when quite dark, and discovered the patient, aged 35 years, in a very emaciated condition, and with intense agony depicted in her countenance. Upon examination, I discovered an immense tumor occupying the greater part of the right iliac region, dipping deeply into the adjacent pelvic cavity, where it had formed permanent adhesion. In its most central part the tumor, when manipulated, manifested unequivocal fluctuation, and attenuated integument. On a cursory examination, and without extending my manipulations beyond the more prominent part of the tumor, my first impression was, that the patient was the victim of an abscess of an enormously enlarged liver. However, before proceeding any farther, I made inquiries of my patient concerning the history of her past and present health and condition, particularly as bearing on this abdominal enlargement. Living in a malarious district of country, she had, for a series of years preceeding, been subject to frequent and repeated attacks of Intermittents, which, from neglect, had given rise to a variety of ailments, such as are usually attendant upon Ague, and the constant introduction of miasm. That, in the meantime, her spleen became very much enlarged and painful---or, as she denominated it, her "ague cake" grew large and tender to the touch; and, that during this time, she had resorted to no remedial means, save such as have been handed down from family to family, and by reason of their traditional value, as having been always sacredly entrusted to the care and transmission of some female or sybil asclepiad, have in consequence become hallowed. That, about a year ago---dating from my visit---she had exerted herself more than was her wont, and in consequence, she felt the "ague cake" to fall from its natural position into the right side. This seemed rather astounding to me, having never in the course of my reading, or personal observation, known such a displacement. I could scarcely give credence to her statement, inasmuch as we often are the witnesses of the excessive credulity and marvellousness of a certain class of females, to which this lady belonged. Nothing loth, I extended my investigation of the case, and discovered, and could distinctly trace, the transverse and a portion of the descending colon, as having actually taken possession of the place once occupied by the spleen, and the spleen displacing a portion of the contents of the right pelvic region, had, by inducing adhesive inflammation, taken possession of the same. That there should be no doubt in my diagnosis, I traced the entire margin of the liver, as likewise that of the now diseased structure, save so much of it as adhered to the upper and inner aspect of the ilium; and furthermore, by deep pressure between the liver and enlarged mass, the lower contour and anatomical unevenness of the former organ was distinctly perceptible, so that I could no longer doubt.

Now I will not pretend to say, nor can I believe that the spleen was suddenly torn from its connection with the stomach, omentum, kidney, &c., but, that by the gradual extension of its connecting media to the forementioned parts, it did, after the violence it first sustained, by the force of its own weight, find the pelvic and iliac localities, where, in all probability, it became as an extraneous body, inasmuch as its connecting vessels had been obliterated. That it could not have sought suddenly the iliac region, is evident from the fact that rupture, if not entire severance of its bloodvessels must have obtained, which must have given rise to fatal hemorrhage, either from the quantity of fluid so thrown out, or from its inducing peritoneal inflammation of an erysipelatous character, if I may be allowed the expression, as used in the sense of John

Hunter, since the patient was eminently in a condition to be subject to such a form of inflammation---she being a miasmatic cachectic. From such an inflammation she, however, did not suffer, as I had every reason to know.

I informed the woman that she could by no possibility ever be restored to even ordinary health; that the only palliation would be the external evacuation of the contents of the abscess; to which she readily assented; and I accordingly proceeded to plunge an abscess lancet into the most suitable point, giving exit to a large quantity of rather healthy pus; which continued to flow pretty freely for six or eight weeks following, during which time I did not see her; neither could I gain permission of a post-mortem examination, which I very much regreted.

In a physiological point of view, I deem this case an interesting one, in deducing conclusions with reference to the functional importance of the spleen in the animal economy. I regret that I could not have had an opportunity to see this patient at an earlier period, and frequently, so as to have been enabled to note closely, the effects on the system of so strange a phenomenon. I, however, satisfied myself that the functions of digestion and assimilation suffered materially, and that my opinion, however novel it may appear, with reference to the spleen, as entertained many years since by an eminent French pathologist, was strengthened. The opinion to which I refer, is the following:—

This pathologian contended he had proven, by dissection carefully conducted, that the spleen was a great nervous secreting organ, analagous to the brain; that, as there are two separate and distinct systems of nerves in all vertebrated animals—the first being designated as the automatic system, or as I have elsewhere observed, the nutritive, ganglionic, or vital system, as being that which controls the involuntary or spontaneous motions—the second, the cerebral, as controlling the voluntary motions; that, as the first system is composed of the great sympathetic, its branches, plexus and ganglia, having especial cognizance of the nutritive and vital functions, he asserted the spleen was an additamentum to the ganglionic system; that it was actually the brain of the abdomen, whose function it was, by the aid of its adjuncts, the ganglia, to secrete that portion of the nervous fluid necessary toward the maintenance of healthy abdominal functious. Hence, if this theory be a true one, how very important and essentially necessary toward a healthy nutrition, a healthy and unimpaired condition of this abdominal brain. That in disease of the spleen, or its total destruction or removal from the body, the abdominal intelligence suffers proportionally. This is a novel theory of the function of the spleen, and because novel, and in opposition to the generally received crude and unsatisfactory opinions of physiologists, may be discarded as one unworthy an investigation.

Prof. J. H. Miller, who wrote on this subject many years ago, says: "The spleen being invariably found in all the higher classes of animals, is abundant evidence of its performing some important duty." Its substance is more soft than other of the abdominal viscera---is more feeble than any substance in the body, unless it be the brain. It is co-existent with the brain, and those animals which are devoid of brain, likewise want the spleen; and that wherever there is a spleen, there, likewise, is a brain; the inference is legitimate, thereforefore, that an intimate relationship exists between the two viscera.

In the language of Prof. M., "the spleen has no marked connection with the cerebral or spinal nerves, but is identified with the nerves of the spontaneous system as its cerebral organ, therefore, any affinity which may subsist between those organs, must depend upon this system of nerves. Now, as those nerves had a prior existence to the brain and its nerves, and were the grand agents in their formation, and continue to be the same of their sustentation, the simultaneous appearance of the spleen and brain can be accounted for only upon the supposition, that the former is appended to the spontaneous system of nerves, to aid it in the supererogatory duty of forming and sustaining the brain and its appendages. That the spleen performs like offices for the primary system, to what the brain does for the cerebral and spinal, is inferred from a similarity of structure.

In conclusion, the following plain and acknowledged facts may be noted. Congestion or engorgement of the spleen, invariably follows repeated paroxysms of the Ague, as well as being consequent npon a residence in a marshy district, without the necessary Ague following.

Periodicity is of a nervous origin; Quinine is the most powerful anti-periodic agent we possess; that in proper doses it becomes a sedative; and that sedation seeks the nervous system for its manifestation; and, finally, Quinine is successfully resorted to in the treatment of splenetic derangement, &c. May these facts not materially aid us in investigating the funciions of the spleen, and its relation to the brain, if any such relation exist. It is at least worthy some attention.

---

[It is due to our esteemed correspondent to say that the above article has been on our table for more than a year. The extreme singularity of the case induced us to postpone its publication, until further correspondence with the author. Although no post mortem examination was had, he assures usthere was no posibility of his diagnos isbeing a mistaken one, and we do not presume to dispute his opinion. We therefore publish the communication as a contribution to the curiosities of medical experience in malarious regions. As for the speculations touching the functions of the spleen, our friend has the satisfaotion of knowing that if his opinions are fanciful, they are like all other hypotheses on this subject.—Eds.]

